# Apps targeting anorexia nervosa in young people: a systematic review of active ingredients

**DOI:** 10.1093/tbm/ibad003

**Published:** 2023-02-08

**Authors:** Clara P Chiang, Daniel Hayes, Elena Panagiotopoulou

**Affiliations:** Research Department of Clinical, Educational & Health Psychology, University College London (UCL), London, UK; Education & Training, Anna Freud National Centre for Children and Families, London, UK; Research Department of Clinical, Educational & Health Psychology, University College London (UCL), London, UK; Evidence Based Practice Unit, UCL and the Anna Freud National Centre for Children and Families (AFNCCF), London, UK; Research Department of Behavioural Science and Health, Institute of Epidemiology & Health Care, University College London, London WC1E 7HB, UK; Research Department of Clinical, Educational & Health Psychology, University College London (UCL), London, UK; Education & Training, Anna Freud National Centre for Children and Families, London, UK

**Keywords:** Anorexia nervosa, mHealth, Behavior change techniques

## Abstract

Evaluating the presence of behavior change techniques (BCTs) in mHealth apps could be used to better understand what “active ingredients” contribute to outcomes. Despite the early onset of Anorexia Nervosa (AN) and the increasing use of mobile apps to seek mental healthcare among young people, BCTs underpinning mHealth apps targeting AN have never been systematically examined. This review systematically identified and analyzed BCTs underpinning apps targeted at reducing AN in young people in an attempt to understand their active components. Apps were searched and screened in Apple Store and Google Play. Six apps that met the inclusion criteria and were coded by trained researchers against the BCT Taxonomy V1. Overall, 22 of 93 possible BCTs were identified. The most common were “Information about health consequences,” “Social support (unspecified),” and “Information about antecedents”. Identified BCTs suggested potential overlaps with traditional clinical treatments for AN, such as cognitive behavioral therapy and family-based therapy. Further investigation is required to evaluate the apps’ usability and effectiveness.

## Teaser

Behavioral change techniques identified in mobile health apps targeting Anorexia Nervosa in adolescence.

**Implications**

- Overall, 22 Behavior Change Techniques have been identified within apps for helping manage Anorexia Nervosa (AN.)
- Overall, the primary interventions proposed in the AN apps tend to be more of an informative nature, focusing on how to reduce unwanted behaviors, rather than focus on promoting healthy and desirable behaviors.
- Future work needs to be undertaken to understand the apps effectiveness on help managing symptoms of AN.

**Practice:** Informs mental healthcare professionals about the most frequently used BCTs within apps for helping manage Anorexia Nervosa (AN), and how they can be incorporated in interventions.

**Policy:** Demonstrates the need for collaboration between clinicians, health behaviour experts and app developers to create apps that include effective BCTs and theory.

**Research:** Opens new avenues for further research into the usability and effectiveness of those apps in managing symptoms of AN.

## INTRODUCTION

In 2021, approximately 70 million people worldwide were affected by eating disorders (ED) [1]. One of the most common ED is Anorexia Nervosa (AN), a disorder characterized by an intense fear of gaining weight [2]. The disorder is also linked to engaging in restrictive-eating behaviors (e.g., fasting, dieting, and excessive exercising), resulting in abnormally low body weight [3]. Another common ED is Bulimia Nervosa (BN), which is characterized by binge-purging eating behaviors (e.g., excessive eating episodes are followed by repeated inappropriate purging behaviors such as misusing laxatives or self-induced vomiting) but results in a relatively normal body weight [2, 3]. Of the UK population affected by ED, approximately 8%–10% suffer from AN while another 40% suffer from BN [4]. However, a study revealed one-third of patients with an initial diagnosis of AN crossed over to BN within a 7-year follow-up period; movement from BN to AN was found to be less common [5]. In line with this, a meta-analysis of 36 studies revealed that 20%–50% of individuals diagnosed with AN developed BN over time [6]. Hence, individuals with BN at the initial point of diagnosis could have previously suffered from AN, suggesting the population affected by AN is actually larger than estimated.

AN typically develops around puberty [7] and peaks predominantly in females aged between 10 and 14 years old [8]. Developing the illness during critical adolescence years not only stunts normal development but also increases the risk of other medical complications. The restricted eating behavior causes severe malnutrition and weight loss, which adversely affects nearly every system within the body [9]. Many medical conditions associated with AN are reversible (e.g., gastroparesis), but some effects can remain permanent (e.g., osteoporosis or decreased fertility due to prolonged amenorrhea) [9]. Moreover, AN often coexists with other psychiatric conditions such as mood and anxiety disorders, depression, trauma-related disorders, and obsessive-compulsive disorders [10]. AN is the most fatal mental disorder with a mortality rate of 10% [11, 12]. Given the young age at which AN develops and the subsequent medical implications, there is a compelling need for the disorder to be diagnosed and treated early.

Established AN recovery programs often employ a multidisciplinary approach. In non-life-threatening situations, interventions involve a combination of talking therapy and supervised weight gain treatments [13]. For adolescents, family-based therapy (FBT) or cognitive behavioral therapy (CBT) is recommended [10]. FBT is based on the concept that the ED belongs to the entire family and the parents’ involvement is necessary for treatment success [14]. Unlike FBT, CBT views the ED as belonging to the individual and treatment success requires the patient to be willing and actively involved in the process of change [14]. Other aspects of the treatment may include building social support networks or working with other mental health professionals and dieticians to set weight restoration goals and plan behavioral corrective strategies [15]. However, a multi-center study in the UK reported that despite weight gain (with a large effect size), most patients when discharged continued to remain highly symptomatic and severely underweight [16]. Moreover, the AN relapse rate in established interventions has been found to reach up to 52% [17].

The recovery process is complex, and a number of factors can lead to relapse and treatment resistance. AN is ego-syntonic by nature, where individuals find aspects of the disorder (i.e., weight control and being thin) valuable and attractive [18–21]. Patients become fearful of “losing” the illness, which often develops as a mechanism to manage unwanted emotions, cope with adverse experiences [22–24], and satisfy a need for control [25]. The feeling of having absolute control over their eating behavior and weight loss is in itself a positive psychological reinforcement to sustain the disorder [26]. Therapy attempts to change the AN behavior can trigger negative feelings and give rise to a sense of losing control [24], thereby activating the coping mechanism. Stigmatization toward individuals with AN is also widespread across the general public [27, 28], health professionals [29, 30], and the individuals suffering from the illness themselves [31]. Chandra and Minkovitz [32] found that the greatest barrier preventing care for adolescents was associated with stigma and those with less mental health knowledge had increased stigmatized views. AN sufferers also experience shame and guilt, which further provokes the need for control [33]. Becker et al. [20], demonstrated that stigma, social costs, and shame are common barriers to seek help across both ethnic minority and nonminority groups. In addition, the shortage of mental health systems worldwide [34] and high financial barriers often prevent people from accessing treatment. Hence, stigma, feelings of shame and guilt, economic and social costs, as well as the ego-syntonic nature of the illness interact in complex ways that compound the adverse impacts and resistance to traditional treatments. It is, therefore, important to consider other forms of treatment to support AN to address the challenges mentioned.

Ownership of smartphones has grown from 1.86 billion in 2015 to 3.6 billion in 2020 [35] and mobile apps are developing at tremendous rates. In 2017, a total of 324,000 mHealth apps were identified across major app stores [36]. In a public survey, 76% of 525 respondents indicated that they would be inclined to use their mobile phone to self-manage their mental health if services were free [37]. They would also be 11 times more likely to self-initiate seeking help online and via mHealth apps compared to conventional in-person professional care [38]. Among the younger population, mHealth apps are popular owing to their ability to overcome the stigma inherent in seeking help for mental health issues [39]. Other advantages of mHealth apps include constant availability, easier access, cost reduction, immediate support, anonymity, and increased clinical service capacity [40]. The small but growing literature on mHealth apps targeting ED suggests not only the potential of these interventions but also the need to assess their validity before implementing them on a larger scale in clinical practice [41]. Anastasiadou et al. systematically reviewed the existing evidence of mHealth interventions for ED and found improvements at post-assessment for mobile apps, vodcasts, and text-messaging tools [41]. Another clinical case study found that a non-commercial self-monitoring app based on CBT principles for treating EDs was well-received by both patients and clinicians, illustrating its potential benefits for transdiagnostic clinical use [42]. However, a recent review of smartphone applications found that ED apps contained few evidence-based components and were not empirically tested for treatment efficacy [43]. Hence, while mHealth apps appear to have the potential to deliver evidence-based treatments and be integrated into conventional clinical treatments [44], the majority of them have not been evaluated for quality and effectiveness.

Evaluating the presence of behavior change techniques (BCTs) in mHealth apps could be used to better understand what “active ingredients” contribute to outcomes [45], which could potentially lend insights into the apps’ quality and effectiveness. A BCT is defined as “an observable, replicable and irreducible component of an intervention designed to alter or redirect causal processes that regulate behavior” [46]. In other words, it is a “common language” adopted by practitioners and researchers to determine the triggers of behavior change [46]. To do this, the BCT taxonomy (v1.0) can be used, which is a constructive, ­international, and multidisciplinary method to evaluate the underpinning principles of behavior change interventions [46]. The taxonomy contains a total of 93 distinct behavior techniques hierarchically organized into 16 clusters, which are used to highlight potential identifiers of motivation, opportunity, interaction, and capacity [46]. While this is the most comprehensive taxonomy available, not all 93 BCTs have equal effectiveness in treating AN. For this reason, various discipline-specific taxonomies were developed, such as the ‘Coventry, Aberdeen & London—Refined (CALO-RE) taxonomy,^1^ which evaluates physical activity and weight interventions [47]. Leonidas and colleagues used the CALO-RE taxonomy to identify BCTs present in traditional treatment manuals developed for AN, including FBT and CBT [49]. The most common BTCs found in FBT included: “Prompt rewards contingent on effort or progress towards behavior,” “Provide rewards contingent on successful behavior,” “Model/demonstrate the behavior,” and “Plan social support/social change.” The most common BCTs found in CBT included: “Prompt review of behavioural goals,” “Prompt self-monitoring of behavior,” “Provide feedback on performance,” and “Prompt practice.” Overall, BCTs that included planning of goals, actions, tasks, and working with motivation were present in both manuals. However, despite the trending usage of mobile technology to promote healthy lifestyles and positive well-being, only few studies have evaluated the BCTs underpinning mental health apps such as self-harm [50] and alcohol addiction [51].

Despite the early onset of AN and the increasing use of mobile apps to seek mental healthcare (mHealth apps) among young people, the BCTs underpinning mHealth apps targeting AN have not been systematically examined. The aim of this study was to systematically identify that BCTs are present in AN apps for young people in an attempt to understand their active components. Although both commercial and non-commercial mental health apps exist and have been previously included in reviews, this review will focus on commercial (free) mHealth apps because they are widely accessible to young people who, as mentioned above, are more likely to self-initiate seeking help for EDs via mHealth apps if services are free.

## Method

### Search Strategy for App Sample

Apps were systematically searched during the period of December 2020 and February 2021 on two platforms: Google Play and the Apple App Store (“Apple Store”). Search terms included “anorexia,” “anorexia nervosa,” “anorexia disorder,” “anorexia recovery,” “anorexia help,” “anorexia intervention,” and “anorexia recovery”. See Table 1 for inclusion criteria:

**Table 1:** Inclusion criteria outlined for this study

Inclusion criteria	Rationale
Apps available for free download on mobile phones	Apps easily accessible by individuals who own a smartphone (Android or Apple).
Apps available in English	Common language amongst all reviewers involved.
Apps available in the Apple Store and/or Google Play	Google Play (2.7 million apps) and Apple Store (1.82 million apps) are the two largest app platforms available in the market [52]
Apps specifically designed for AN	The review focuses on apps targeting AN and not other EDs.
Apps designed for use by individuals with AN	The review focuses on apps targeted at individuals with AN and not their social support network (e.g., friends, parents, mental health professionals, etc.).
Apps designed for use as an intervention or relapse prevention tool	The review focused on apps used as an intervention or relapse prevention tool and not as diagnostic tools (e.g., quizzes to identify the type of eating disorder an individual is experiencing) and general diet trackers.
Apps with sufficient information to extract BCTs	The function of some apps was solely to link users to support groups/ chats/ health professionals and, therefore, did not contain sufficient detail to code BCTs.
Apps designed for children and young people (0–24 years)	According to United Nations (n.d.), “youth” is defined as people aged between 15 and 24 years old and “children” are people aged 14 years old and below.
Apps with unique content	Some apps have identical content so the app with the earliest published date was selected.

### Screening and Selection Process

**Figure 1:**
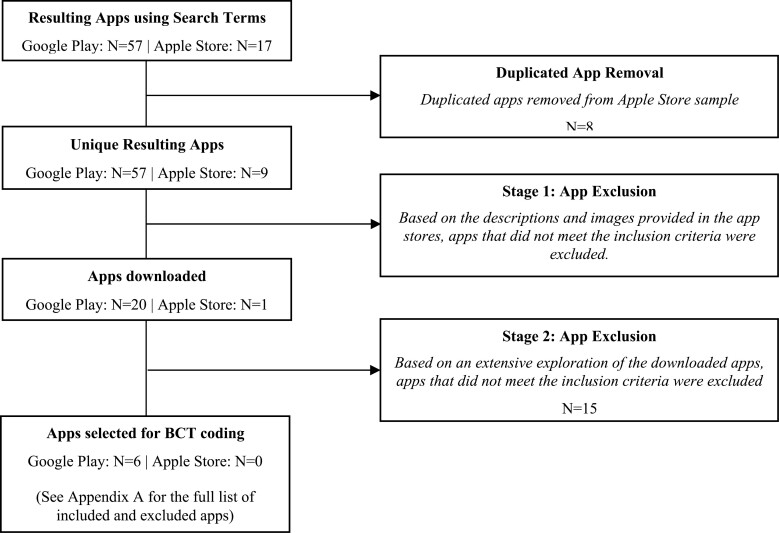
Flow diagram of app screening and selection for BCT coding.

The screening and selection of apps were carried out in two stages and involved two reviewers. In stage one, the search terms were entered in Google Play and Apple Store. Duplicates were removed from the Apple Store list and only considered in the Google Play list. With reference to the inclusion criteria (Table 1), Researcher A independently screened the apps via descriptions and images available in the app store. Any apps that were identified as potentially suitable were downloaded. In stage 2, downloaded apps were screened again by both Researcher A and Researcher B, who jointly examined the apps’ content more extensively with reference to the inclusion criteria. Some apps downloaded in stage 1 did not meet all the inclusion criteria in stage 2 and were excluded from this review. Involving a second researcher helped reduce any biases and/or oversight from Researcher A. Figure 1

### BCT Coding

All apps that met the inclusion criteria were accessed using the same devices throughout the evaluation (Samsung Galaxy S10 or iPhone X). Test devices were unmodified smartphones that had the latest up-to-date versions of operating systems installed. The entire content of each app was coded to extract BCTs, which included data from texts and images. Apps were downloaded and coded once. The developer of each app was contacted to determine whether they consulted a health industry expert when developing the app and whether the app had undergone any form of evaluation, yet there were no responses. Hence, the full BCT taxonomy (v 1.0) [46], comprised of 93 techniques, was used to evaluate the apps. This taxonomy was chosen to ensure all techniques present in the apps were captured, including those that may not be specific to treating AN. The process of coding mHealth apps consisted of two stages. The first stage was carried out independently by Researcher A who extracted and coded BCTs. Data collected were recorded electronically, including: (1) the excerpt or image that has been coded, (2) the identified BCT, (3) the researcher’s confidence level of the given BCT, annotated with + (semi-confident) or ++ (confident), and (4) rationale justifying the coding. The second stage was a multi-code review, where the data collected by Researcher A was reviewed by Researcher B. With reference to the BCT taxonomy, the task of Researcher B was to check for errors in the extracted excepts and identified BCTs. Any discrepancies found in the codes were resolved through discussions between Researcher A and B. However, if the two researchers were unable to come to an agreement, a third reviewer, “Researcher C,” who has considerable experience in behavior change was involved in the discussions. Once a unanimous agreement on the coded BCTs was reached, each unique BCT and its frequency of usage in each mHealth app were recorded. All three reviewers undertook and completed the BCT taxonomy training (https://www.bct-taxonomy.com/) prior to this study, which is an online course comprised of six modules and two assessments.

### Synthesis of Results

The results synthesized from the selected mHealth apps were presented in a narrative form. Basic descriptive statistics were calculated and graphs and tables were also incorporated to clearly illustrate the unique BCT techniques and their hierarchical clusters employed across all the apps and within each app.

## Results

The general characteristics of selected apps are outlined below in Table 2.

**Table 2:** General characteristics of included apps

App name (*developer*)	Behaviour/ED targeted	Language	Free/Paid	Age	No. of reviews	Review rating out of 5.0	No. of downloads
**Diet & Help for Anorexia** (*Kaveri Tyag*i)	anorexia	English	Free	3+	19	3.8	1000+
**Anorexia Calendar** (*App Diggity LLC*)^a^	anorexia	English	Free	3+	5	3.4	1000+
**Anorexia** (*Dintale*)	anorexia	English	Free	3+	12	3.2	1000+
**Anorexia Recover** (*Dintale*)	anorexia	English	Free	3+	n/a	n/a	500+
**Anorexia Recovery** Guide (*Jackline Moline*)	anorexia	English	Free	3+	n/a	n/a	500+
**Natural Treatments for Anorexia** (*RK Unit*)	anorexia	English	Free	3+	n/a	n/a	n/a

*Note.* All apps in this table are available in Google Play.

^a^App(s) also available in Apple Store.

Overall, of the 93 possible BCTs, a total of 22 BCTs (23.6%) were identified. In total, there were 120 instances of BCTs across the apps. The most commonly used BCT was “Information about health consequences” (27 instances, 22.5%), followed by “Social support (unspecified)” (24 instances, 20.0%), “Information about antecedents” (23 instances, 19.2%) and “Instruction on how to perform the behaviour” (13 instances, 10.8%). These four BCTs accounted for 72.5% (87 instances) of the total instances of BCTs founds. The only BCT that was used consistently across all six apps was “Social support (unspecified).” The remaining 18 out of 22 BCTs were used less than 5 times, of which 10 BCTs occurred on one instance (0.8%). See Appendix B for a list of all the unique BCTs, definitions, coded excerpt examples, total instances (frequency) and frequency percentage across the six apps.

**Figure 2:**
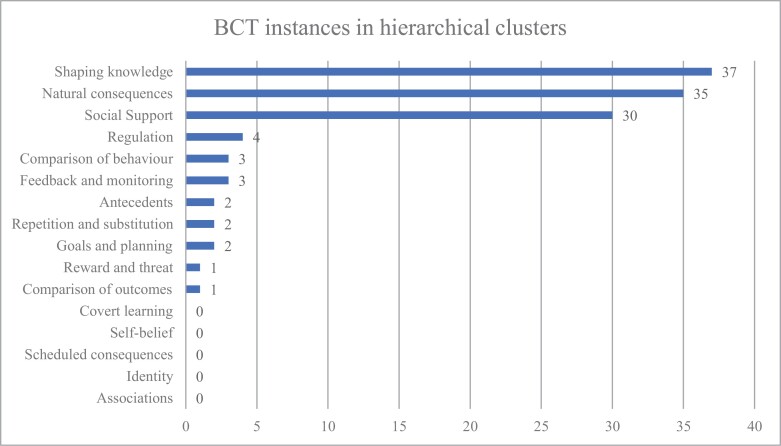
BCTs organized into hierarchical clusters.

When BCT instances were organized into their hierarchical clusters, the BCTs identified belonged to 11 (68.8%) out of the 16 clusters. The top three clusters were “Shaping knowledge” (37 instances, 30.8%), “Natural consequences” (35 instances, 29.2%), and “Social support” (30 instances, 25.0%), which made up 85.0% of all the BCT instances. The five clusters not utilized in any of the apps were “Covert learning,” “Self-belief,” “Scheduled consequences,” “Identity,” and “Associations.” Figure 2 displays the total tally of BCT instances when organized into their BCT hierarchical clusters.

The variation of unique BCTs across the six apps ranged between 3 and 11 BCTs. *Anorexia Recovery Guide* had a total of 11 unique BCTs and *Anorexia Recover* had a total of 3 unique BCTs. See Table 3 for the number of unique BCTs used in each of the six apps. The number of BCT instances that appeared in each app ranged between 7 and 38 BCTs. *Natural Treatments for Anorexia* had a total of 38 BCT instances with “Information about antecedents” occurring 42.1% (16 instances) and “Social support (unspecified)” occurring 26.3% (10 instances). *Diet & Help for Anorexia* had the second highest frequency with 27 BCT instances, of which “Information about health consequences” occurred 44.4% (12 instances) and “Information about antecedents” occurred 22.2% (6 instances). *Anorexia Calendar* had the lowest frequency of 7 BCT instances of which “Social support (unspecified)” occurred 42.9% (3 instances). See Table 3 for the total number of BCT instances that occurred in each app.

**Table 3:** Number of BCTs used and frequency of BCT instances in each app

App name	Number of unique BCTs used	Total BCT instances
Anorexia recovery guide	11	25
Natural treatments for anorexia	9	38
Diet & help for anorexia	7	27
Anorexia	5	14
Anorexia calendar	5	7
Anorexia recover	3	9

The majority of the extracted excerpts within these apps were coded with one BCT. However, in a few instances, coded content included a combination of BCTs. Specifically, the combination of “Information about health consequences” and “Salience of consequences” occurred three times in *Diet & Help for Anorexia*. The combination of “Instruction on how to perform a behaviour” and “Action planning” occurred twice in *Anorexia Recovery Guide.* Finally, the combination of “Pharmacological support” and “Social support (unspecified)” emerged in two apps, *Anorexia Recover* and *Natural Treatments for Anorexia*.

## Discussion

The aim of this review was to systematically identify and analyze the BCTs present in mHealth apps targeted at young people with AN. Six apps met the inclusion criteria and they were all available on Google Play, whereas only one of them was available on Apple store, suggesting that the accessibility of iPhone users to mHealth apps is more limited. A total of 22 (23.6%) out of 93 unique BCTs were identified across the six apps and the most frequent were “Information about health consequences,” followed by “Social support (unspecified)” and “Information about antecedents.” The BCTs identified more frequently can provide some insight into the content of the apps and allow the exploration of how this may align with evidence-based treatments. For instance, the BCTs “Information about health consequences” and “Information about antecedents” seem to place emphasis on helping AN individuals to be more aware of the negative consequences, as well as situations that reliably predict unwanted behaviors. However, due to the disorder’s ego-syntonic nature, such knowledge could potentially have limited impact. “Social support (unspecified)” was the second most common BCT used in apps suggesting that battling AN requires the support of others—a network of friends, family, and health experts. Overall, the primary interventions proposed in AN apps tend to be more of an informative nature, focusing on how to reduce unwanted behaviors, yet there is a lack of focus on interventions that promote healthy and desirable behavior.

Nevertheless, the hierarchical clusters of identified BCTs revealed some consistencies between app interventions and traditional clinical approaches. The identified BCTs were spread across 11 out of the 16 different hierarchical clusters in the taxonomy, of which the majority (85.0%) of BCTs were categorized into “Shaping knowledge,” “Natural consequences,” and “Social support.” These clusters are in line with some of the treatment aspects of FBT and CBT recommended in the NICE guidelines [53]. “Shaping knowledge” and “Natural consequences” are both BCT clusters consistent with the CBT approach, providing individuals with a better understanding and knowledge of how to manage their illness. The “Social support” cluster involves the support and encouragement from friends, family, and professional experts, which is strongly consistent with FBT. To a degree “Social support” is also aligned with CBT, which views the role of family as useful (e.g., to help the individual in implementing new behaviors), yet not crucial [54]. This is further supported by Leonidas et al.’s [49] study that identified aspects of social support were more prevalent in FBT than CBT. The ­remaining 15% of BCTs present were included in clusters such as “Reward and threat,” “Goals and planning,” and “Feedback and monitoring” but these were only employed on one, two and three instances, respectively. These clusters, however, contain BCTs that traditional clinical approaches would consider essential to the implementation of AN behavioral changes. For example, FBT and CBT often require working with mental health professionals and dieticians to set weight restoration goals and plan behavioral corrective strategies, which are monitored on a regular basis to track progress [15]. This is also supported by Leonidas et al. [49] who identified “rewards” as active ingredients in FBT and “goals and feedback” as active ingredients in CBT. The minimal use of such BCT clusters suggests that the apps are lacking compared to traditional clinical approaches, such as FBT and CBT, in ­techniques that involve planning of goals and actions, as well as working with motivation [49].

While approximately two-thirds of the clusters were present across the apps, there were some clusters that were not utilized at all, such as “Associations,” “Scheduled consequences,” and “Identity.” Some of these BCTs could potentially promote AN behavioral change. For example, within the “Identity” cluster, “Framing/reframing” helps individuals adopt a new perspective to change cognitions or emotions related to a behavior [46], which is also a core aspect of CBT (e.g., address negative thoughts about their body image, weight, and eating) [15]. However, since app developers did not comment on whether health industry experts were consulted when developing the apps, it is not clear why these BCTs were excluded.

This study identified BCTs in mHealth apps targeted and AN in young people, however, it does not capture the effectiveness of these BCTs in helping treat AN. Review ratings of the apps could be an indicator of effectiveness, but this is not a reliable proxy. Three out of the six apps had review ratings, but each rating was based on the average of less than 20 reviews, and it is uncertain what criteria (e.g., functionality, user experience, information quality, engagement, etc.) the ratings were based upon. Hence, little can be drawn from the review ratings with regards to potential impact apps have on helping young people manage their AN. There have been studies that evaluate the use and effectiveness of mHealth apps for other disorders such as anxiety and depression [55], but none on AN mHealth apps. Therefore, it is recommended that further research with app users is conducted to test the usability and effectiveness of mental health apps, as well as to evaluate which BCTs promote behaviors to improve AN in young people. This will help improve the quality of mHealth apps and provide healthcare professionals with the knowledge to advise on the usage of such apps.

This is the first study to review the BCTs underpinning AN mHealth apps targeted at young people, using a comprehensive taxonomy of BCTs [46], developed though an international consensus process. This study can help researchers and app developers gain insight into the techniques employed within existing apps, as well as determine the direction of future studies to improve content quality of apps. Moreover, this study is wide reaching, as it evaluated apps that are freely accessibly on Google Play and Apple Store, which are the two largest app platforms available in the market today [52]. Another strength of this study is the comprehensive and systematic approach taken to screen and select apps, collect data, and analyze content. The review was completed with two independent reviewers and if there were any disagreements, a third expert reviewer was involved, which is a standard practice for systematic reviews [56]. The involvement of three independent researchers helped reduce the risk of systematic bias and inaccuracies in the extraction of BCTs.

The study also possesses some limitations. The apps included were limited to being free and available in the English language. It is possible that other apps may have employed other BCTs that were not found in this study and/or may have been based upon relevant theories and ­clinical approaches in conjunction with health industry experts. Also, although measures were put in place to reduce the risk of systematic bias and inaccuracies, is possible that the some information provided in the apps (such as videos) made some aspects of coding slightly more subjective.

To conclude, the findings of this review suggest that several of the techniques employed in apps, belonging to the hierarchical clusters of “Shaping knowledge,” “Natural consequences,” and “Social support,” are consistent with those embedded in traditional clinical approaches (i.e., FBT and CBT). However, other techniques focusing on planning of actions, goals, tasks, and working with motivation are missing. Subsequent investigations using the same BCT taxonomy to evaluate mHealth apps and evidence-based treatments would be valuable to ascertain the extent to which they are aligned. This can potentially help determine how mHealth apps could be effectively used adjunct to traditional interventions. Further research is required to evaluate and improve the usability and effectiveness of mHealth apps for AN, as well as to determine which specific BCTs have more impact on, and appeal to, the younger population. With the growing popularity of technology and mHealth apps amongst the younger population, researchers and healthcare professionals should work closely together with app developers. This will help to bridge the gap between technology and mental health treatments to develop more effective apps that will help the younger population with behavior change. App developers may also consider developing apps targeting specific eating behaviors common in different EDs, as well as increasing the mHealth app options for iPhone users in order to reach the wider population.

## Footnotes

1 The CALO-RE taxonomy comprises of 40 distinct BCTs with no official hierarchical clusters [47]. It was derived based on the first cross-behavior BCT taxonomy developed by Abraham and Michie [48], which was more recently revised to the BCT taxonomy (v1.0) containing 93 distinct BCTs [46].

## Appendix A

**Table:**

*Apps included* * in this study * (*N* = 6)		
**Google Play** Anorexia Nervosa Abnormal Eating Disorder & Habits (Kaveri Tyagi)	Anorexia Nervosa Help Calendar (App Diggity LLC) Anorexia (Dintale) Anorexia Recovery (Dintale)	Anorexia Recovery Guide (Jackline moline) Natural Treatments for Anorexia (RK Unit)
***Duplicated apps*** * that were removed from the Apple Store list and kept in the Google Play list *(*N* = 8)		
Anorexia Nervosa Help Calendar RR Eating Disorder Management Brighter Bite – ED Recovery	Rise Up: Eating Disorder Help Eating Disorder Test Recovery Record for Clinicians	Feeleat Love your Kite
***Excluded apps in Stage 1*** * of the screening and selection process * **(*N* = 45)**		
Anorexia & Friendship Ate Food Diary Better Help Binge Eating Disorders BMI Calculator BodyFast Intermittent Fasting Tracker	Eating Disorder Recovery Eating Disorder Test Eating Disorders Eating Disorders Guide EatingHabits Fasting App Feeleat	Mental Illness Assessment Tests Metabolic Compass MyFast Track NanoCurso Anorexia y Bulimia Overeaters Anonymous Speaker Psychopedia Recovery Record for Clinicians
Bulimia Nervosa Help Calendar Calorie Counter by FatSecret Casual Dieting— Weight Manager DBT Coach deVicer Dining Note Eat Breathe Thrive Eat Right Now Eat This Much	Food Addiction Advice Food Diary Healthy Diet How to Stop Binge Eating Effectively Islam and Eating Disorders Leave Emotional Eating Behind LIFE Intermittent Fasting Love you Kite	Rise Up + Recover RO DBT RR: Eating Disorder Management See How You Eat Food Diary Treat Eating Disorders Weight Diary Wysa Zero Calories Fasting Tracker
***Excluded apps in Stage 2*** * of the screening and selection process * **(*N* = 15)**		
Anorexia and Amenorrhea Anorexia and Neurobiology Anorexia Effect’s Danger Anorexia Highest Mortality Atypical Anorexia Nervosa Blue Buddy	Brighter Bite—ED Recovery Eating Disorder Guide Eating Disorders Home Remedies for Anorexia (*Ajfreebiz*) Home Remedies for Anorexia (*Tith Kongmeng*)	Long-term Effects of Anorexia Natural Remedies for Anorexia (*Raja Jawad Ahmed*) Refeeding Anorexia Patients Remedies for Anorexia

***Note.*** For apps with replicated content of another app, the app with the earliest published date was taken into consideration for this study

## Appendix B. Unique BCTs, definitions, examples, frequency, and frequency percentage across the six apps.

**Table:**

	BCT	Definition	Coded excerpt example	Frequency (number of instances)	Frequency (%)
1.	Information about health consequences	“Provide information (e.g., written, verbal, visual) about health consequences of performing the behaviour”	“Anorexia can cause severe physical problems because of the effects of starvation on the body. It can lead to loss of muscle strength and reduced bone strength”	27	22.5%
2.	Social support (unspecified)	“Advise on, arrange or provide” social support (e.g., from friends, relatives, colleagues,’ buddies’ or staff) or noncontingent praise or reward for performance of the behaviour. It includes encouragement and counselling”	“Get counselling … in particular, look into cognitive behavioural therapy (CBT) … a CB therapist will help to break the patterns of disordered eating through the use of food monitoring, thought monitoring and meal regularity and nutritional monitoring”	24	20.0%
3.	Information about antecedents	“Provide information about antecedents (e.g., social and environmental situations and events, emotions, cognitions) that reliably predict performance of the behaviour”	“Predicting factors of anorexia include social pressure to be thin, difficulty expressing feelings, a lack of social or family support…”	23	19.2%
4.	Instruction on how to perform the behavior	“Advise or agree on how to perform the behaviour (includes ‘Skills training’)”	“Advise on eating a small or medium serving of a nutrient-dense food provides needed calories and nutrition”	13	10.8%
5.	Social support (practical)	“Advise on, arrange, or provide practical help (e.g., from friends, relatives, colleagues, ‘buddies’ or staff) for performance of the behaviour”	“A physician should be supervising the recovery process and meeting with you in a medical office on a regular basis … weekly weigh-ins, vital signs measurement, and periodic laboratory testing including CBC, serum electrolytes and serum amylase levels”	5	4.2%
6.	Information about social and environmental consequences	“Provide information (e.g., written, verbal, visual) about social and environmental consequences of performing the behaviour”	“The illness can affect people’s relationship with family and friends, causing them to withdraw”	3	2.5%
7.	Pharmacological support	“Provide, or encourage the use of or adherence to, drugs to facilitate behaviour change”	“Advise to be on life-long calcium, have normal levels of vitamin D (or take supplements to achieve normal levels)”	3	2.5%
8.	Salience of consequence	“Use methods specifically designed to emphasise the consequences of performing the behaviour with the aim of making them more memorable (goes beyond informing about consequences)”	“Complications of anorexia includes dehydration and can lead to highly concentrated urine and more urine production (image provided to emphasise the consequence)”	3	2.5%
9.	Social comparison	“Draw attention to others’ performance to allow comparison with the person’s own performance”	“Learn from others, seek out success stories from other people recovering from anorexia … figure out what they did to change their relationship with food and eating for the better”	3	2.5%
10.	Action planning	“Prompt detailed planning of performance of the behaviour (must include at least one of context, frequency, duration and intensity)”	“Eat throughout the day … eat regular meals, spaced about three to four hours apart”	2	1.7%
11.	Habit formation	“Prompt rehearsal and repetition of the behaviour in the same context repeatedly so that the context elicits the behaviour”	“Snack more frequently. Reminding yourself to eat more often, to snack between meal and to eat whenever you feel hungry … get into the habit of snacking throughout the day on small healthy foods”	2	1.7%
12.	Self-monitoring of behaviour	“Establish a method for the person to monitor and record their behaviour(s) as part of a behaviour change strategy”	“Keep a food journal. Keeping track of food intake can lead to healthier eating habits”	2	1.7%
13.	Adding objects to the environment	“Add objects to the environment in order to facilitate performance of the behaviour”	“Keep a small scale and measuring cups on hand when preparing meals”	1	0.8%
14.	Conserving mental resources	“Advise on ways of minimising demands on mental resources to facilitate behaviour change”	“Advise to measure and weigh your food, humans are not a good judge of size, so keep a small scale and measuring cups on hand when preparing meals”	1	0.8%
15.	Credible Source	“Present verbal or visual communication from a credible source in favour of or against the behaviour”	“I suffered from the age of 12 until I finally sought help at the age of 24 … I thought that change would never be possible and therapy was such hard work. It took a long time but I eventually entered recovery and have never looked back. My life now is wonderful!”	1	0.8%
16.	Distraction	“Advise or arrange to use an alternative focus for attention to avoid triggers for unwanted behaviour”	“Distract yourself with these unique and incredible images (series of photos were provided)”	1	0.8%
17.	Feedback on behaviour	“Monitor and provide informative or evaluative feedback on performance of the behaviour (e.g., form, frequency, duration, intensity)”	“Time you have saved so far # Days, # Hours # Minutes” (a time that clocks the period you refrain from engaging in anorexic behaviour)	1	0.8%
18.	Information about emotional consequences	“Provide information (e.g., written, verbal, visual) about emotional consequences of performing the behaviour”	“What it is like to have anorexia … As I lost weight I began to feel tired and this made me more depressed, I couldn’t think straight or concentrate in school … I realise now I was suffering from the effects of starvation”	1	0.8%
19.	Monitoring emotional consequences	“Prompt assessment of feelings after attempts at performing the behaviour”	“Track how you’re feeling before and after you eat, and what kind of thoughts you’re having that may affect eating habits and lead to unnecessary food restriction”	1	0.8%
20.	Non specific reward	“Arrange delivery of a reward if and only if there has been effort and/or progress in performing the behaviour (includes ‘Positive reinforcement’)”	“Trophies are locked until your progress passes each phase. You are then awarded trophies to help you stay motivated and tract your progress”	1	0.8%
21.	Re-attribution	“Elicit perceived causes of behaviour and suggest alternative explanations (e.g., external or internal and stable or unstable)”	“Though the restrictive eating patterns that characterise this anorexic eating disorder are similar to dieting behaviours, there are stark differences … while someone may diet in an attempt to control weight, anorexia nervosa is often an attempt to gain control over one’s life and emotions”	1	0.8%
22.	Social support (emotional)	“Advise on, arrange, or provide emotional social support (e.g., from friends, relatives, colleagues, ‘buddies’ or staff) for performance of the behaviour”	“Advise on setting goals with your doctor, mental health professional and registered dietitian [and set] goals in the area of learning emotional self-care and developing trust in people who are trying to help you”	1	0.8%
			**Total**	**120**	**100.0%**

## TRANSPARENCY STATEMENTS

**Study registration:** This study was part of an MSc project and was registered with the dissertation module lead.

**Analytic plan registration:** The analytic plan was reviewed by the applicants’ supervisor and module lead.

**Availability of data:** Data from this review is available via request from the study authors.

**Availability of analytic code:** Descriptive analysis and coding from this review is available via request from the study authors.

**Availability of materials:** There are no materials associated with this review.

**Teaser**: 22 active ingredients in apps targeting AN were identified, some which overlap with traditional forms of psychotherapeutic treatment.
